# On Concurrent Scarlet Fever and Measles

**Published:** 1856-10

**Authors:** W. B. Kesteven


### Papers and Communications.

### ON CONCURRENT SCARLET FEVER AND MEASLES.

By W. B. KESTEVEN, Esq., F.R.C.S.

If eruptive fevers always presented precisely the characters attributed to them in books, we might have cause to wonder that our forefathers should have experienced any difficulty in distinguishing between them. We should be justified in expressing amazement that Rhazes found it necessary to be very explicit in drawing the distinction between small-pox and measles—a distinction drawn so faithfully, that the practitioner of the present day may discover therein a picture of the difficulties that he has most probably himself encountered at the bedside. We might be somewhat incredulous when told that Sir Robert Sibbald hesitated to give an opinion upon cases of epidemic Scarlatina in Edinburgh in 1680; we might feel at a loss to account for the great renown that was, nevertheless deservedly, accorded to Dr Fothergill for having distinctly traced the affinities of an epidemic Scarlatina Anginosa; we should have had some reason for expressing distrust of the acuteness of apprehension of those among our ancestors who could have confounded measles, small-pox, and scarlatina.

That this regularity, or uniformity, however, does not exist in nature, a not very long experience will soon discover to the practitioner. Difficulties of diagnosis have, within a period of the last three or four years, frequently happened to the author in regard to scarlatina and measles. His number of cases, however, not having been sufficiently great to serve the purpose of any useful statistical comparison, the writer has contented himself with stating the general features of the cases to which he refers, as illustrated by the subjoined (as they may be termed) representative cases.

In the month of September of this year the writer attended five children in a house in Albany Street, Regent's Park. In each case there was indisposition for four or five days, consisting of simple febrile or catarrhal symptoms, with cough, coryza, watering and redness of the eyes. On the fourth or fifth day the severity of the symptoms abated coincidently with the appearance of an eruption of a dull reddish colour, not, to the sense of touch, elevated above the surrounding cuticle; when viewed through a lens about a quarter of an inch distance, many of its apices appeared to be desquamating on the second day. At first it looked very like the early eruption of confluent small-pox, but wanting the raised hard and granular feeling to the finger of that disease. The eruption was scattered over the body and limbs. On the face, neck, chest, and arms, it presented the irregular shaped clusters seen in measles. The tongue was furred, and its apex was as red as a boiled lobster. The throat, in three instances was slightly sore, and was of a scarlet colour. The pulse was rapid, but soft. The bowels rather costive. Urine scanty, but not high coloured; and free from albumen.

These were the most prominent features observed. The variations were, that in two of the children the eruption was more distinct, the spaces of clear skin between the spots being of greater extent. Head symptoms were seen in one child, subsiding with the fever and eruption. The eruption continued apparent for from three to five days, and then rapidly disappeared; the cuticle subsequently desquamating as in scarlatina. An interval of a fortnight occurred between the subsidence of the disease in three of these children, and the first appearance of the symptoms in the two youngest. A period of two days only intervened between each case of the three eldest. Some of the children in this family had been to a school where several of their schoolfellows were absent on account of illness—said to be measles. The author had himself attended the elder children in measles two or three years previously.

Irregularity, very similar to that described in the preceding cases, as already stated, has been met with on many occasions during the last three or four years. In some cases the first symptoms have corresponded less to those of measles than above related.

The cases here recorded are regarded by the writer as furnishing instances of the concurrence of measles and scarlatina, the symptoms respectively of each of these fevers masking those of the other, and causing embarrassment to the diagnosis. They have, in most instances, presented a striking resemblance to the description of measles given in his *Practice of Medicine* by Cullen as that met with in his day; while they possessed also the undoubted characteristics of scarlatina.

This conjunction of eruptive fevers, if admitted as the correct view of these cases, presents an exception to the principle so emphatically laid down by John Hunter, and to a very great extent adopted by his successors, that two different fevers cannot exist in the same constitution at the same time. There is little doubt, however, that this principle cannot be accepted rigidly and without modification. Many parallel instances have been cited by Mr. Marson from his own experience at the Small-Pox Hospital, and communicated to the Royal Medico-Chirurgical Society in a paper read May 26th, 1847. "Thus", concludes Mr. Marson, "either from personal observation, or from the writings of others, I present examples of the simultaneous occurrence of variola and scarlatina, variola and rubeola, variola and pertussis, variola and vaccinia, rubeola and scarlatina, rubeola and vaccinia, rubeola and pertussis, varicella and vaccinia, pertussis and vaccinia."

Rhazes, as already observed, in his *Treatise upon Small-Pox*, bestows great pains in establishing the diagnosis of that disease from measles, and labours to show that Galen had also accurately distinguished between them In the words of Dr. Montgomery,* "the most remarkable inaccurracy prevailed in former days on this subject (the diagnosis of eruptive fevers), since we find Sennertus, in the middle of the seventeenth century, discussing the question 'why the disease, in some constitutions, assumed the form of small-pox, and in others that of measles;' and in a posthumous work of Diemerbroeck, published in 1687, it is laid down that small-pox and measles are only different degrees of the same affection. The same doctrine was still more recently maintained by Langè, a professor at Leipsic."

Such having been the confused state of the diagnosis of measles and small-pox, we should be prepared to find no less confusion between measles and scarlatina. This is to be observed in the names under which the latter has been known, *e.g.*, morbilli confluentes, rubeola rosalia, febris rubra, enarthesis rosalia. Although Sydenham had completely established the differences between small-pox and measles, the latter and scarlatina continued to be regarded as varieties of the same fever. So gradually indeed did the distinction become recognised, that it is not known by whom the word Scarlatina was first employed.

Dr. Montgomery (*Cyclopædia of Practical Medicine, Art.* "*Rubeola*") observes "in our country Morton maintained the identity of measles and scarlatina, and considered the relation existing between them the same as that between distinct and confluent small-pox. Even so recently as 1769, Sir William Watson confounded these two diseases, the cor-

---

* Cyclopædia of Practical Medicine.

rect diagnosis of which ought probably to be referred to the time of publishing the second edition of Dr. Withering's *Essay on Scarlet Fever* in 1793." So closely do specific eruptions sometimes run into each other, that Van Swieten, in his commentaries upon Boerhaave's aphorisms, regards measles and scarlet fever as being allied to erysipelas.

The preceding brief review of the history of the diagnosis of these two eruptive fevers, furnishes presumptive evidence that scarlatina and measles must, in former times, have frequently presented a close resemblance in their features; while the cases recorded as of recent occurrence strengthen that evidence, and show that, as the two fevers may coexist, or closely coincide in the period of their appearance, the fact of their so long having been confused under one name by our ancestors must cease to be matter of surprise on our part.*

---

### ON THE OCCURRENCE OF FEVER AT COWBRIDGE, GLAMORGANSHIRE, IN THE AUTUMN OF 1853.

By WILLIAM CAMPS, M.D.; one of the Foreign and Colonial Secretaries of the Epidemiological Society of London.

(*Read before the Epidemiological Society, April* 2, 1855.)

* Since the preceding observations were read at the Epidemiological Society, the attention of the author has been directed to a paper in the *Medical Times and Gazette* (Nov. 6th, 1852) on "Scarlatina Morbillosa (Rubeola)," by his friend Dr. Tripe. In the same journal (Nov. 1852), a case of a like character is reported by W. M. Clarke, Esq., from the Bristol General Hospital. Some correspondence on Epidemic Rubeola has also subsequently been published in the *Lancet* (May, June, July, of the present year), by Dr. Willshire, Dr. Tripe, *Scotus*, and the author. Dr. Willshire's object has been to point out the distinctions between Rubeola and Morbilli on the one hand, and Scarlatina on the other;—a distinction, as observed by Dr. Willshire, previously made by Dr. Copland, and by German pathologists.

